# UBE2T knockdown inhibits gastric cancer progression

**DOI:** 10.18632/oncotarget.15947

**Published:** 2017-03-06

**Authors:** Changjiang Luo, Yunyi Yao, Zeyuan Yu, Huinian Zhou, Lingyun Guo, Junqiang Zhang, Hongtai Cao, Genyuan Zhang, Yumin Li, Zuoyi Jiao

**Affiliations:** ^1^ Department of General Surgery, Lanzhou University Second Hospital and Key Laboratory of Digestive System Tumors of Gansu Province, Lanzhou, Gansu, 730030, China; ^2^ Department of Medical Technology and Key Laboratory of Biotechnology for Laboratory Medicine of Suzhou, Suzhou Vocational Health College, Suzhou, Jiangsu, 215009, China

**Keywords:** UBE2T, ubiquitination, gastric cancer, tumor apoptosis, invasion and metastasis

## Abstract

Ubiquitin-conjugating enzymes (E2 enzymes) such as UBE2T target proteins for degradation via the proteasome. Here, we examined the effects of UBE2T on the progression of gastric cancer. UBE2T was highly expressed in gastric tumors and gastric cancer cells. siRNA-mediated suppression of UBE2T inhibited gastric cancer cell proliferation and colony formation by promoting cell cycle arrest at G2/M phase and increasing apoptosis. Suppression of UBE2T also attenuated the invasive and metastatic abilities of gastric cancer cells by altering expression of epithelial-mesenchymal transition (EMT)-related factors. A xenograft model in which nude mice were injected with UBE2T knockdown human gastric cancer cells confirmed that suppression of UBE2T also decreased tumor formation and growth *in vivo*. Expression levels of CCND1, Phospho-GSK3B, WNT family members, and MYC were all affected by UBE2T knockdown. These results suggest that UBE2T plays a critical role in gastric cancer, and that it may serve as a useful prognostic biomarker and therapeutic target in gastric cancer patients.

## INTRODUCTION

Ubiquitination, including both monoubiquitination and polyubiquitination, plays an important role in proteasome-mediated the protein degradation. The ubiquitination process, which includes activation by ubiquitin-activating enzymes (E1s), conjugation by ubiquitin-conjugating enzymes (E2s), and ligation by ubiquitin ligases (E3s), can alter protein interactions, location, function, activity, and lifespan. It also affects the cell cycle and regulates cancer-related processes, DNA repair, and inflammation [1, 2]. Ubiquitin-conjugating enzymes (E2s), which target proteins for degradation via the proteasome, accept ubiquitin from the E1 complex and catalyze monoubiquitination. They are involved in mitomycin-C (MMC)-induced DNA repair, the association of Fanconi anemia complex with the E3 ubiquitin-protein ligase FANCL, and catalyzing the monoubiquitination of FANCD2 [3, 4]. They also contribute to the ubiquitination and degradation of BRCA1 [5, 6]. Ubiquitin-conjugating enzyme E2T (UBE2T) combines with a specific E3 ubiquitin ligase to induce the degradation of or functional changes in substrate molecules. In addition to its role in Fanconi anemia, UBE2T is increasingly recognized as a critical factor during carcinogenesis in human nasopharyngeal, prostate, breast, and lung cancer [6–8]. However, no systemic studies of UBE2T in gastric cancer that include *in vitro* and *in vivo* models along with clinical samples have yet been conducted.

Gastric cancer, which is the most common type of malignant tumor in China, is associated with distinct regional differences. The incidence of gastric cancer in Gansu province is significantly higher (more than 4-fold) than that of other regions [9]. In addition, the prognosis of gastric cancer is dependent on pathological stage, tumor location, tumor type, biological behavior, and treatment used [10]. In this study, we comprehensively examined the roles, mechanisms, and regulation of UBE2T in gastric cancer for the first time.

## RESULTS

### UBE2T expression was increased in gastric tumors

**Figure 1:**
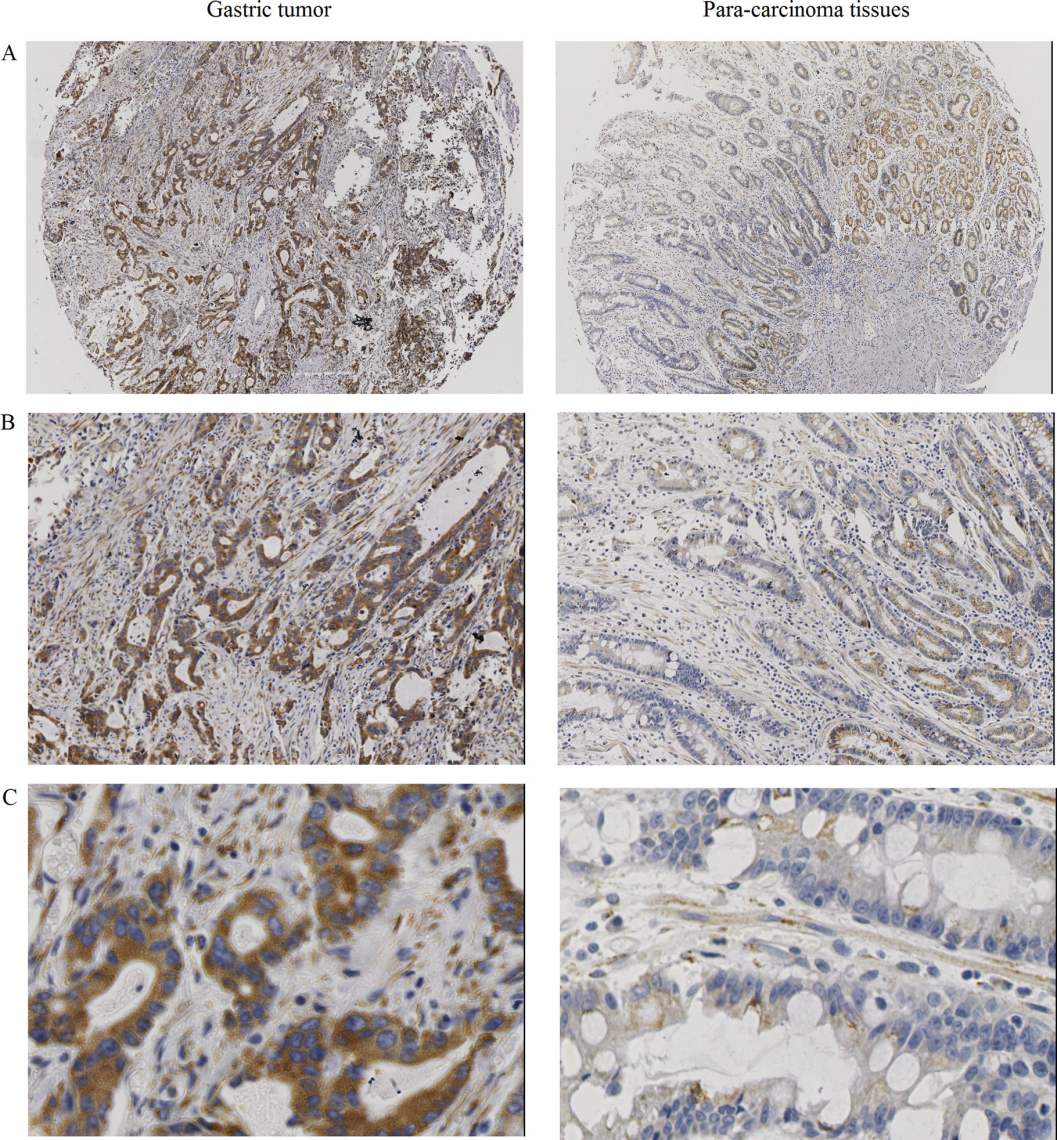
UBE2T protein expression in gastric tumors and para-carcinoma tissues UBE2T expression was increased in gastric tumors compared to para-carcinoma tissues. (**A**) 40 ×, (**B**) 100 ×, (**C**) 400 ×.

To investigate the effects of UBE2T on gastric cancer progression, UBE2T expression was examined in 130 gastric tumor samples and in 39 para-carcinoma tissues using IHC. As shown in Figure 1, UBE2T expression was obviously increased in gastric tumors compared to para-carcinoma tissues (A: 40 ×, B: 100 ×, C: 400 ×). The number of samples with high UBE2T expression was also higher for gastric tumor samples than for para-carcinoma samples (*P* < 0.05, Table 1). UBE2T expression was then examined in samples with different degrees of differentiation; however, no differentiation-dependent differences were observed (*P* > 0.05, Table 2).

**Table 1:** The expressions of UBE2T in gastric tumor samples and para-carcinoma tissues

	Expression of UBE2T Number of samples		Total samples
	Low expression	High expression	
Gastric tumor	113	17	130
Para-carcinoma	39	0	39
Total samples	152	17	169

Note: *P* < 0.05 using both two-sided test and one-tailed test. This result indicates statistical significance between tumor samples and para-carcinoma tissues.

**Table 2:** The expressions of UBE2T in gastric tumors with different degree of differentiation

	Expression of UBE2T Number of samples		Total samples
	Low expression	High expression	
Poorly differentiated	61	7	68
Moderately differentiated	49	9	58
High differentiation	3	1	4
Total samples	113	17	130

Note: χ^2^ = 1.258, *P* = 0.533, *P* > 0.05. This result indicates no significant difference on the differentiation degrees of gastric tumors.

### UBE2T was up-regulated in TCGA database gastric carcinoma samples and widely expressed in malignant gastric cancer cells

**Figure 2:**
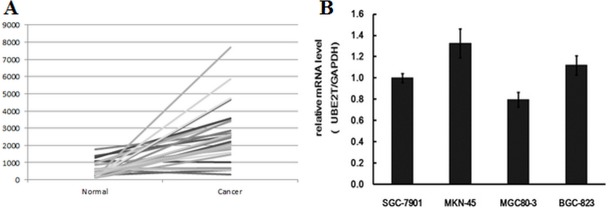
UBE2T mRNA expression in gastric carcinoma tissues and TCGA database samples (**A**) UBE2T expression was increased in gastric tumors in TCGA database. Out of the 29 clinical samples examined, UBE2T expression was also increased in 23 samples, and was unchanged in 6 samples, compared to para-carcinoma tissue; UBE2T expression was not down-regulated in any samples. (**B**) Real-time PCR revealed that UBE2T was widely expressed in SGC-7901, MKN-45, MGC80-3, and BGC-823 human gastric carcinoma cells, and higher expression was associated with more poorly differentiated tumors. Data were analyzed using the 2^−ΔΔCt^ method and are presented as means ± SD. GAPDH was used as a native control and relative expression (UBE2T/GAPDH) was evaluated.

To further investigate the effects of UBE2T on gastric cancer progression, we also analyzed UBE2T expression in the tumor samples in TCGA database. As in the clinical samples, UBE2T expression was higher in tumors than in para-carcinoma tissues in the database. As shown in Figure 2A, among 29 gastric carcinoma tissues, UBE2T gene expression was increased in 23, and unchanged in 6, tumor samples compared to para-carcinoma tissue; no tumor samples exhibited UBE2T down-regulation. UBE2T expression was then measured in different gastric cancer cell types using real-time PCR. GAPDH was used as native control and data were analyzed according to the 2^−ΔΔCt^ method. Relative UBE2T expression (UBE2T/GAPDH) was then evaluated. UBE2T was widely expressed in all of the common human gastric carcinoma cells. UBE2T expression in selected cells is shown in Figure 2B. UBE2T expression was highest in MKN-45 gastric carcinoma cells isolated from poorly-differentiated adenocarcinoma, while MGC80-3 gastric carcinoma cells had the lowest expression. These results indicated UBE2T was up-regulated in most gastric carcinoma tissues and cells, particularly in poorly-differentiated cancer cells.

### siRNA-mediated inhibition of UBE2T in gastric cancer cells

**Figure 3:**
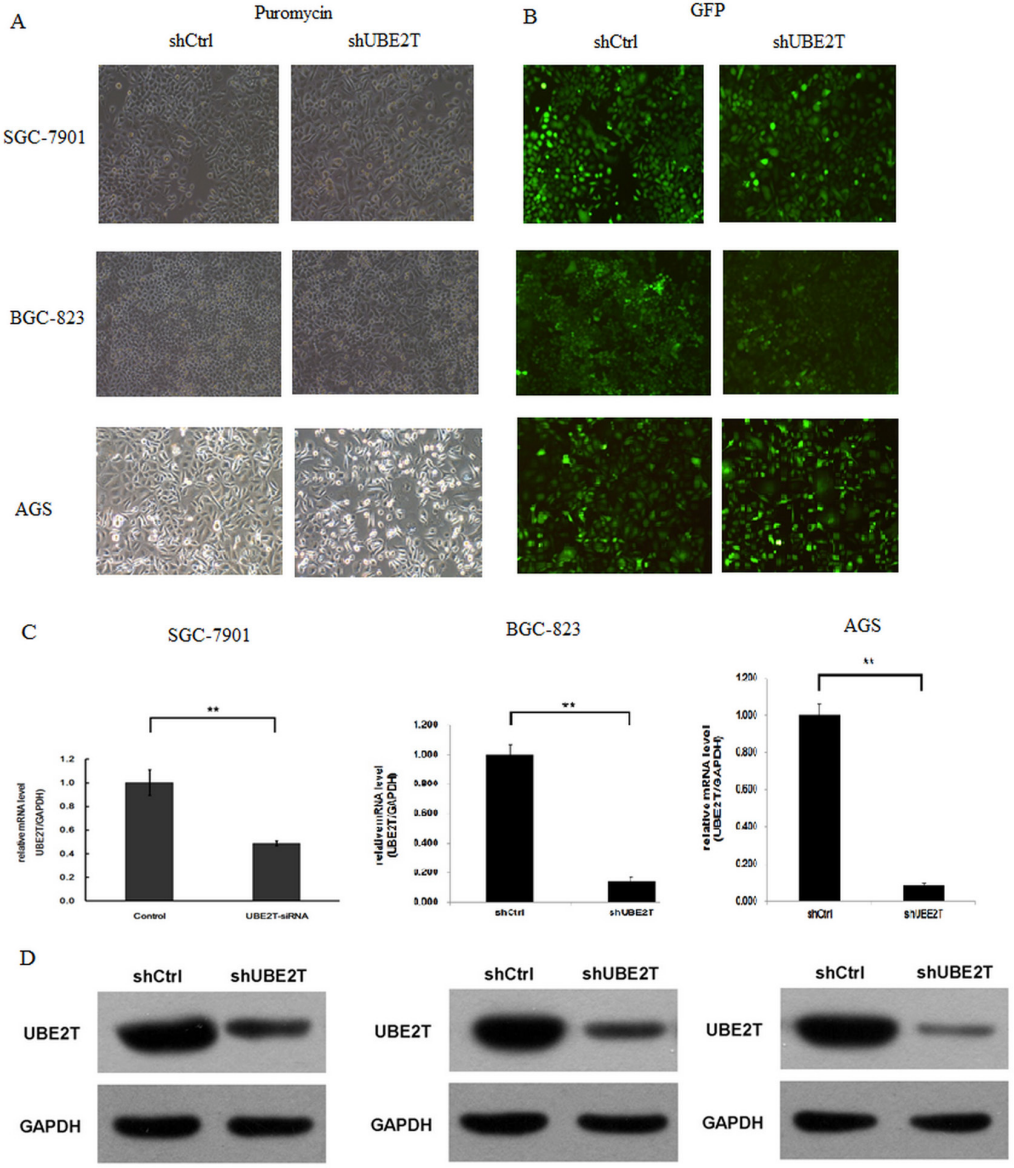
siRNA-mediated inhibition of UBE2T Gastric cancer cells were infected with lentiviruses expressing siRNA targeting UBE2T. (**A**) Puromycin resistance screening for detecting siRNA lentivirus infections in SGC-7901, BGC-823, and AGS cells. (**B**) Green fluorescent protein (GFP) detection of siRNA lentivirus infections in SGC-7901, BGC-823 and AGS cells. (**C**) Real-time PCR assays were performed and relative expression (UBE2T/GAPDH) was evaluated in SGC-7901, BGC-823, and AGS cells. Data are presented as means ± SD. UBE2T mRNA expression was greatly decreased in the shUBE2T groups compared to the shCtrl control groups in SGC-7901, BGC-823, and AGS cells. **P < 0.01 versus control group. (**D**) Western blots for detection of UBE2T. GAPDH was used as native control. UBE2T protein expression was greatly decreased in shUBE2T groups compared to shCtrl groups in SGC-7901, BGC-823, and AGS cells.

Given that increased UBE2T expression is associated with carcinogenesis, we next examined whether suppression of UBE2T could inhibit and attenuate proliferation, invasion, and metastatic abilities in gastric tumors. To that end, siRNA was used to knock down UBE2T expression in three common gastric cancer cell lines: SGC-7901, BGC-823, and AGS. As shown in Figure 3, puromycin resistant screening (A) and green fluorescent protein (GFP) detection (B) revealed that siRNA lentivirus infection was successful in SGC-7901, BGC-823, and AGS cells. UBE2T expression was then measured using real-time PCR. GAPDH was used as a native control and relative UBE2T expression (UBE2T/GAPDH) was evaluated. siRNA infection dramatically decreased UBE2T gene expression in SGC-7901, BGC-823, and AGS cells (*P* < 0.01, Figure 3C). As shown in Figure 3D, Western blots revealed that UBE2T protein expression also dramatically decreased in SGC-7901, BGC-823, and AGS cells after siRNA infection. Cells in which UBE2T expression was inhibited were named shUBE2T, while the native control cells transfected with empty vector were named shCtrl.

### Suppression of UBE2T inhibited growth and colony formation, increased G2/M phase cell cycle arrest, and promoted apoptosis in gastric cancer cells

**Figure 4:**
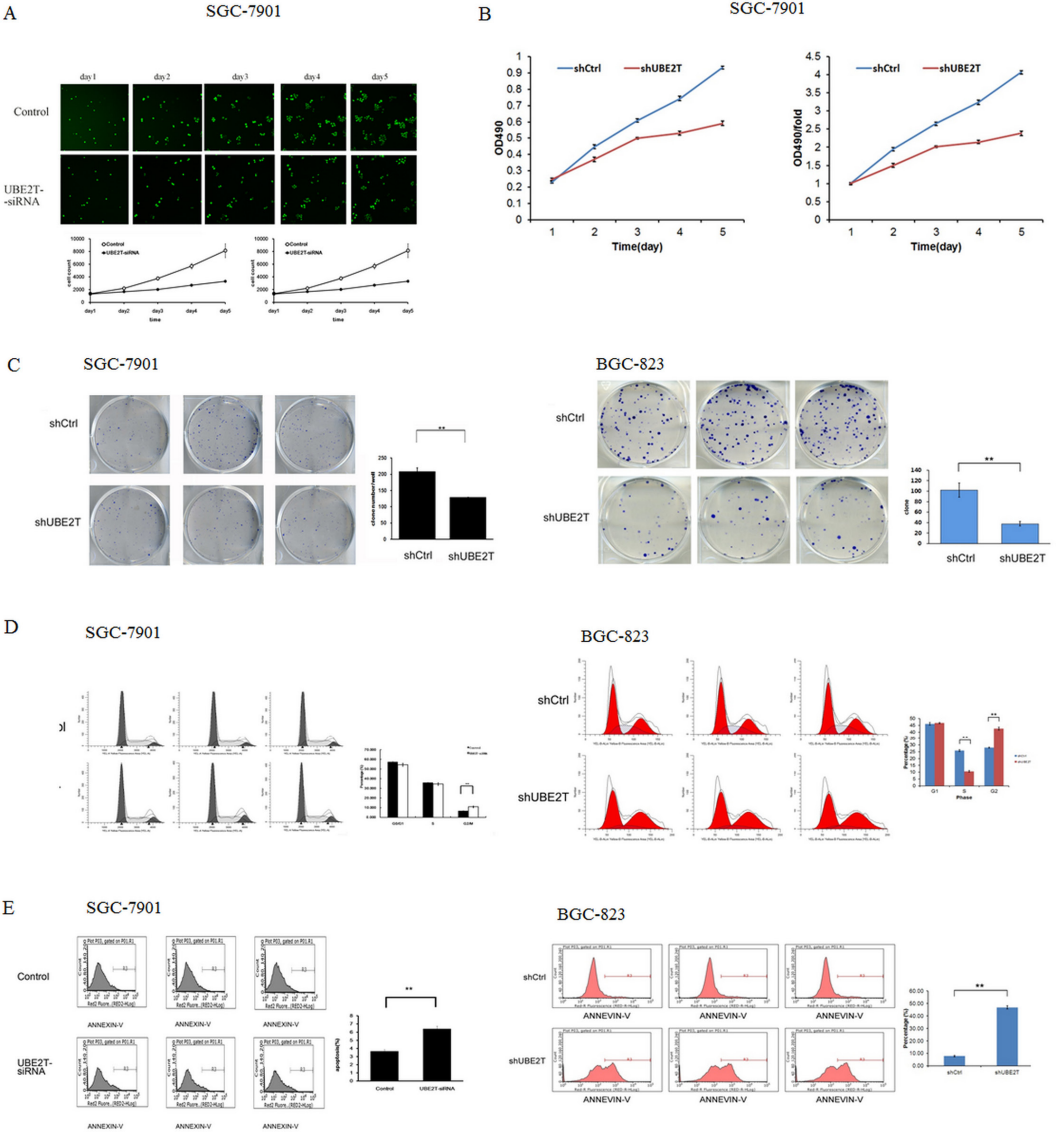
Suppression of UBE2T inhibits growth and colony formation in gastric cancer cells (**A**) Cellomics detection indicated that SGC-7901 cell proliferation decreased after suppression of UBE2T. Data are presented as means ± SD. (**B**) MTT assays in SGC-7901 cells indicated that suppression of UBE2T inhibited SGC-7901 cell proliferation. Data are presented as means ± SD. (**C**) The tumor colony formation assay indicated that suppression of UBE2T inhibited tumor colony formation in SGC-7901 and BGC-823 cells. Data are presented as the means ± SD. **P < 0.01 versus the control group. (**D**) The percentages of cells in the G1, S, and G2/M phases were determined using FCM. G1 and S phase populations decreased while the G2/M phase population increased in SGC-7901 and BGC-823 cells. Data are presented as means ± SD. **P < 0.01 versus the control group. (**E**) Annexin V staining was measured using FCM to evaluate apoptosis. The percentage of apoptotic SGC-7901 and BGC-823 cells increased dramatically after siRNA lentivirus infection. Data are presented as means ± SD. **P < 0.01 versus the control group.

To examine the effects of UBE2T suppression on cancer cell proliferation, cellomics detection and MTT assays were conducted. Cellomics is the discipline of quantitative cell analysis using bioimaging methods. As shown in Figure 4A, after siRNA lentivirus infections, proliferation clearly decreased in SGC-7901 cells. This indicated that UBE2T expression was associated with cell proliferation and that suppression of UBE2T inhibited the proliferation of SGC-7901 cells. MTT detection in SGC-7901 cells produced similar results. As shown in Figure 4B, the OD_490_ value, which is indicative of the number of living SGC-7901 cells, also decreased in a time-dependent manner after siRNA lentivirus infections. MTT detection also indicated that suppression of UBE2T inhibited the proliferation of SGC-7901 cells in a time-dependent manner, reaching maximum inhibition on the 5th day. We then conducted a tumor colony formation assay. As shown in Figure 4C, in both SGC-7901 and BGC-823 cells, the number of colonies was much lower on the 13th day after plating in shUBE2T groups compared to the shCtrl groups (*P* < 0.01), indicating that suppression of UBE2T inhibits colony formation in gastric cancer cells. Flow cytometry (FCM) was then performed to examine the effects of suppression of UBE2T on cell cycle progression. The percentages of shUBE2T cells in the G1 and S phases of the cell cycle were decreased, while the percentage in the G2/M phase was obviously increased, compared to shCtrl cells (*P* < 0.01, Figure 4D). This indicated that down-regulation of UBE2T increased cell cycle arrest at the G2/M phase. Because abnormal cell cycle progression can result in cellular apoptosis, we examined apoptosis in gastric cancer cells using FCM to detect Annexin V staining. As shown in Figure 4E, the percentage of apoptotic cells greatly increased after the siRNA lentivirus infection in both SGC-7901 and BGC-823 cells (*P* < 0.01), indicating that suppression of UBE2T promoted both cell cycle arrest and apoptosis in gastric cancer cells.

### Suppression of UBE2T attenuated the invasive and metastatic abilities of gastric cancer cells

**Figure 5:**
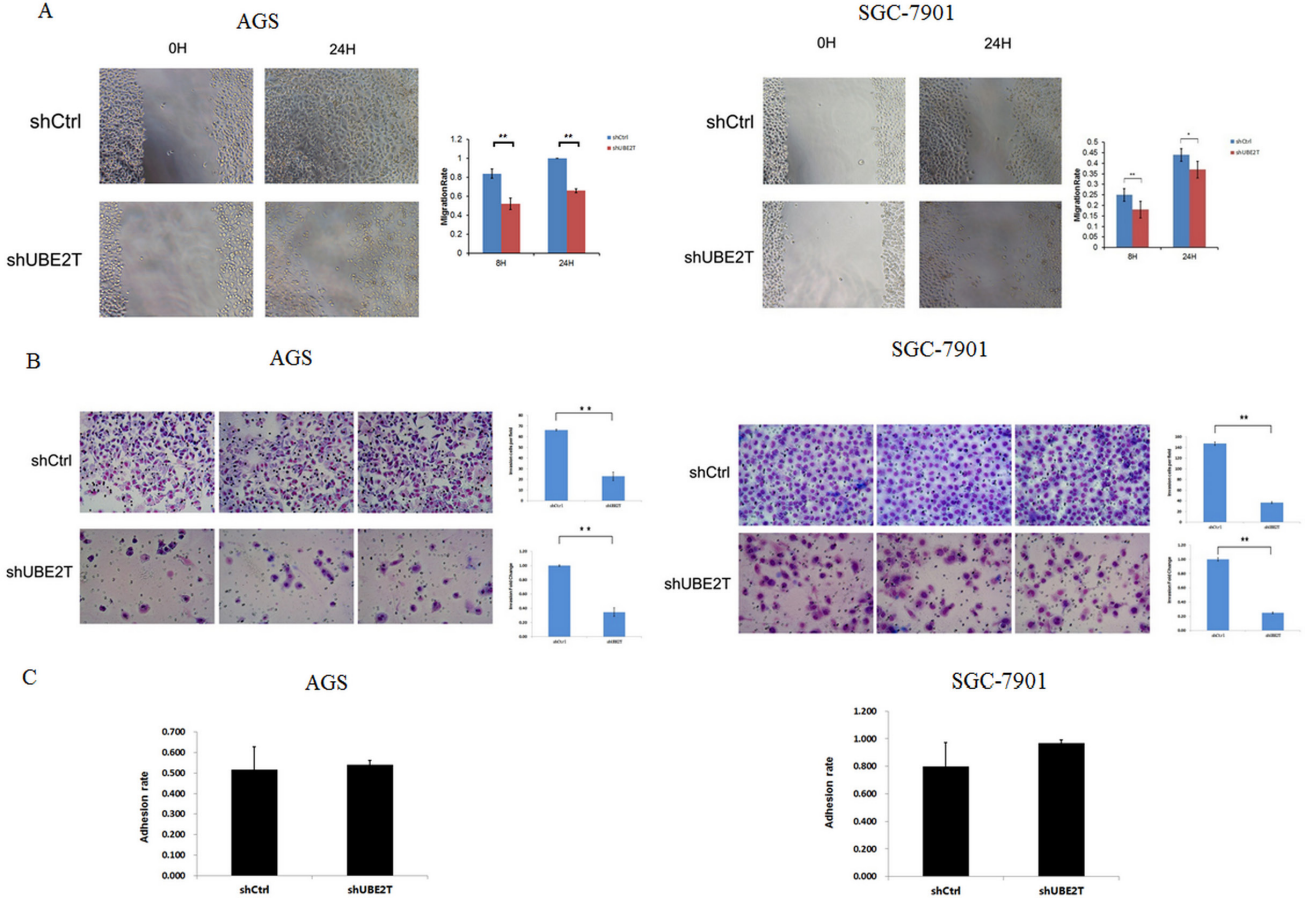
Suppression of UBE2T attenuates invasion and metastasis in gastric cancer cells (**A**) The cell scratch assay was used to examine the invasive and metastatic abilities of gastric cancer cells. Migration rates in the shUBE2T groups decreased after both 8 and 24 h for both AGS and SGC-7901 cells. Data are presented as means ± SD. *P < 0.05 and **P < 0.01 versus the control group. (**B**) The matrix-coated trans-well assay was performed to evaluate the invasive and metastatic abilities of gastric cancer cells. Cell invasion was greatly inhibited in the shUBE2T groups for both AGS and SGC-7901 cells. Data are presented as means ± SD. **P < 0.01 versus the control group. (**C**) The cell adhesion test revealed no differences between the shUBE2T and shCtrl groups for AGS and SGC-7901 cells. Data are presented as means ± SD.

Invasion and metastasis of tumor cells leads to the formation of secondary tumors in other tissues and organs and is the main cause of cancer-related mortality and morbidity. We therefore examined the effects of UBE2T on the invasive and metastatic abilities of AGS and SGC-7901 gastric cancer cells. Suppression of UBE2T attenuated migration and invasion of AGS and SGC-7901 cells in scratch and matrix-coated trans-well assays. As shown in Figure 5A, migration rates in the AGS and SGC-7901 shUBE2T groups were lower at both 8 h and 24 h after attachment compared to shCtrl groups in the cell scratch test (*P* < 0.01; for 24 h after attachment in SGC-7901, *P* < 0.05). Cell invasion was strongly inhibited in both the AGS and SGC-7901 shUBE2T groups compared to the shCtrl groups (*P* < 0.01, Figure 5B). However, there were no obvious differences in cell adhesion rates between the shUBE2T and shCtrl groups for either cell type in the cell adhesion test (*P* > 0.05, Figure 5C).

### Suppression of UBE2T altered expression of epithelial–mesenchymal transition factors

**Figure 6:**
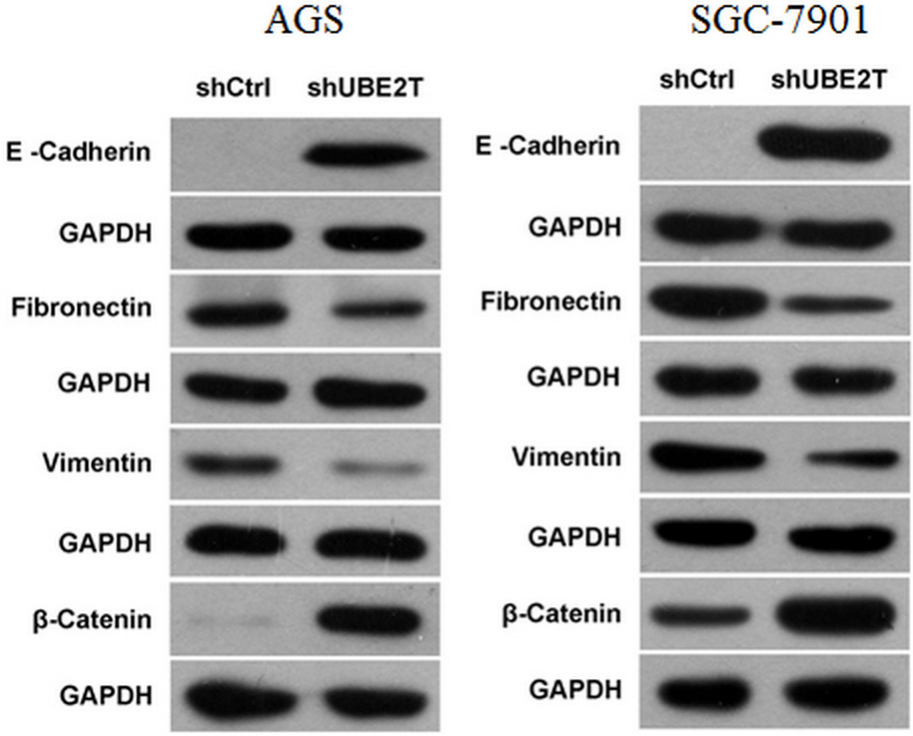
Changes in the expression of EMT-related factors after suppression of UBE2T E-Cadherin and β-Catenin expression increased, while Fibronectin and Vimentin expression decreased, in the shUBE2T groups for both AGS and SGC-7901 cells compared to the shCtrl groups.

Epithelial–mesenchymal transition (EMT), the main regulators of which are E-Cadherin, Fibronectin, Vimentin, and β-Catenin, is a key step in the initiation of cancer metastasis. Western blots revealed that, while E-Cadherin and β-Catenin were hardly detected in both the AGS and SGC-7901 shCtrl groups (except for β-Catenin in SGC-7901), inhibition of UBE2T greatly increased their expression. In contrast, Fibronectin and Vimentin expression were greatly inhibited in both shUBE2T groups compared to the shCtrl groups. Representative images are shown in Figure 6.

### Suppression of UBE2T attenuated tumor formation and growth

**Figure 7:**
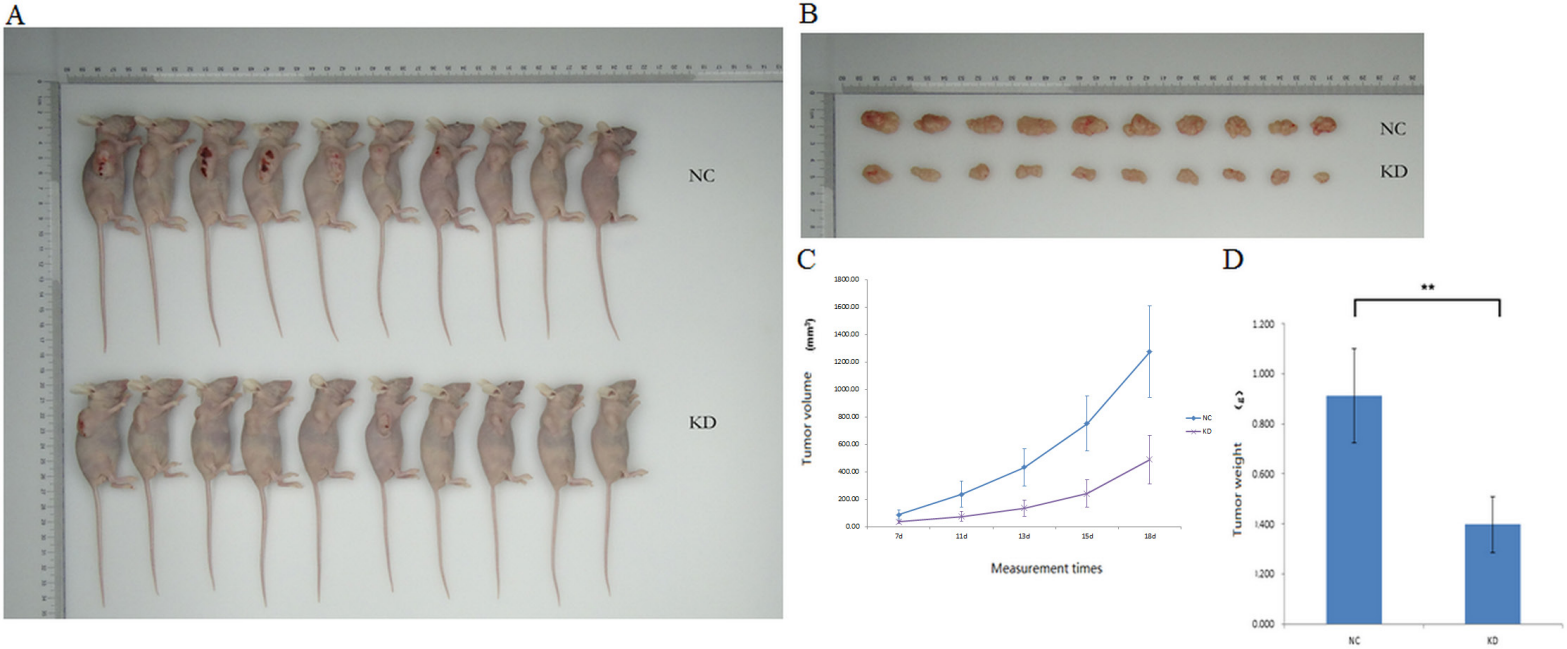
Suppression of UBE2T decreased tumor formation (**A**) Tumor growth was slower in the KD group than in the NC group (*n* = 10 per group). (**B**) Tumors were collected on the 18^th^ day after xenotransplantation. (**C**) Tumor volume curves for mice in the NC and KD groups. Time after xenograft (7d, 11d, 13d, 15d, and 18d) is shown on the x-axis. Data are presented as means ± SD. (**D**) Tumor weights were lower in the KD group than in the NC group. Data are presented as means ± SD. **P < 0.01 versus the control group.

Next, we performed *in vivo* studies to confirm the effects of UBE2T on tumor formation and growth observed in the *in vitro* experiments. We injected siRNA lentivirus-infected BGC-823 gastric cancer cells in which UBE2T expression was greatly inhibited into nude mice to generate the KD group. Ten mice each were assigned to the KD and NC groups. As shown in Figure 7A, tumors grew rapidly in the NC group, indicating that the gastric cancer cells proliferated quickly. In contrast, tumors grew much more slowly in the KD group. Mice were sacrificed on the 18^th^ day after injection and the tumors were collected (Figure 7B). Tumors from the NC group were larger and heavier than those from the KD group (*P* < 0.01, Figure 7C and 7D). Together, these results indicate that suppression of UBET2 inhibited the growth of human gastric carcinoma cells, and consequently tumor formation and proliferation, *in vivo*.

### Path-array analysis for UBE2T

**Figure 8:**
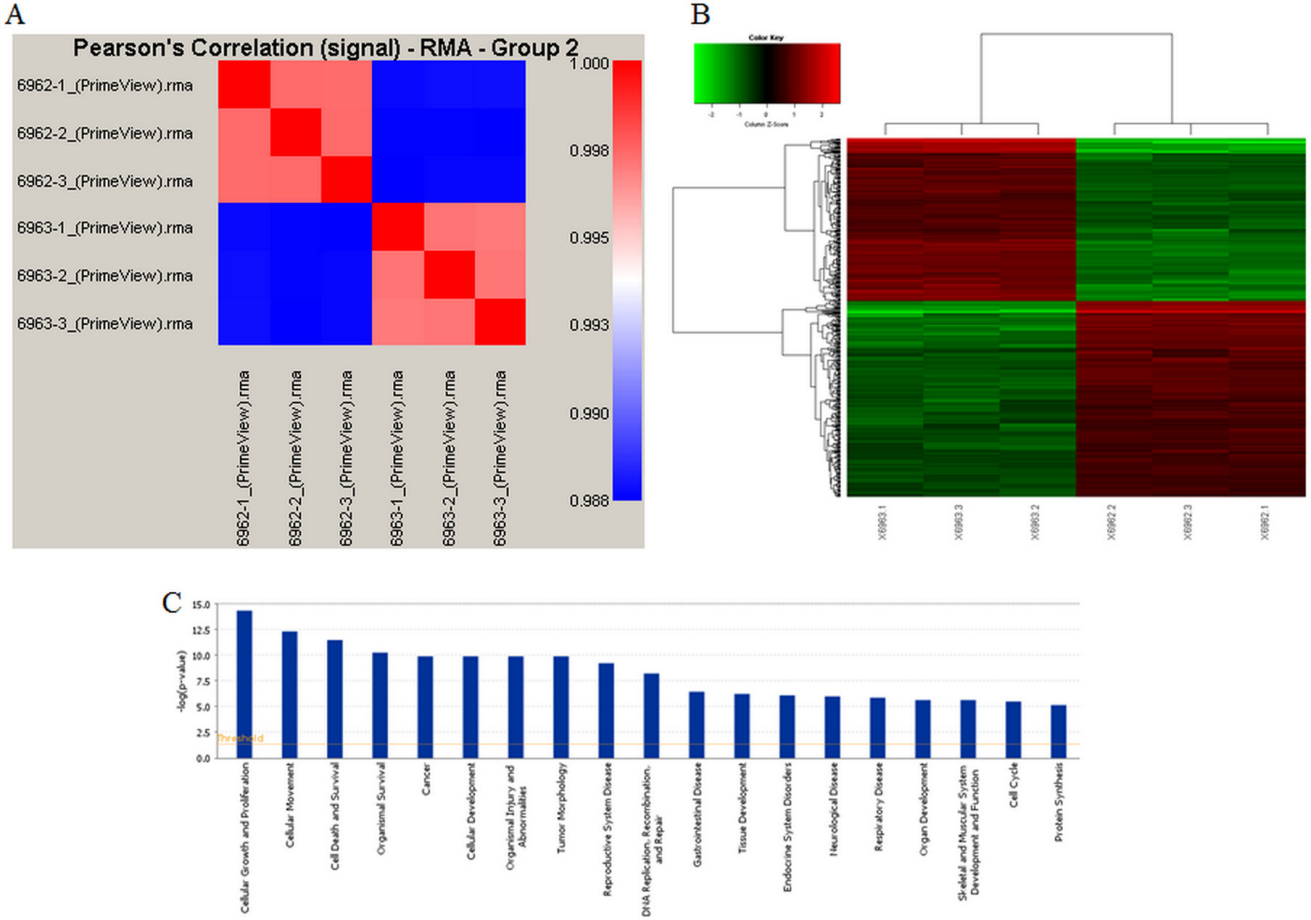
Gene chip analysis of UBE2T regulation (**A**) Correlations between samples in the NC and KD groups were analyzed; Pearson's Correlation Signals are shown. (**B**) The clustering dendrogram shows differences in gene expression between the NC and KD groups. (**C**) Meta-analysis was conducted using IPA online software to identify pathways that might be regulated by or related to UBE2T.

**Figure 9:**
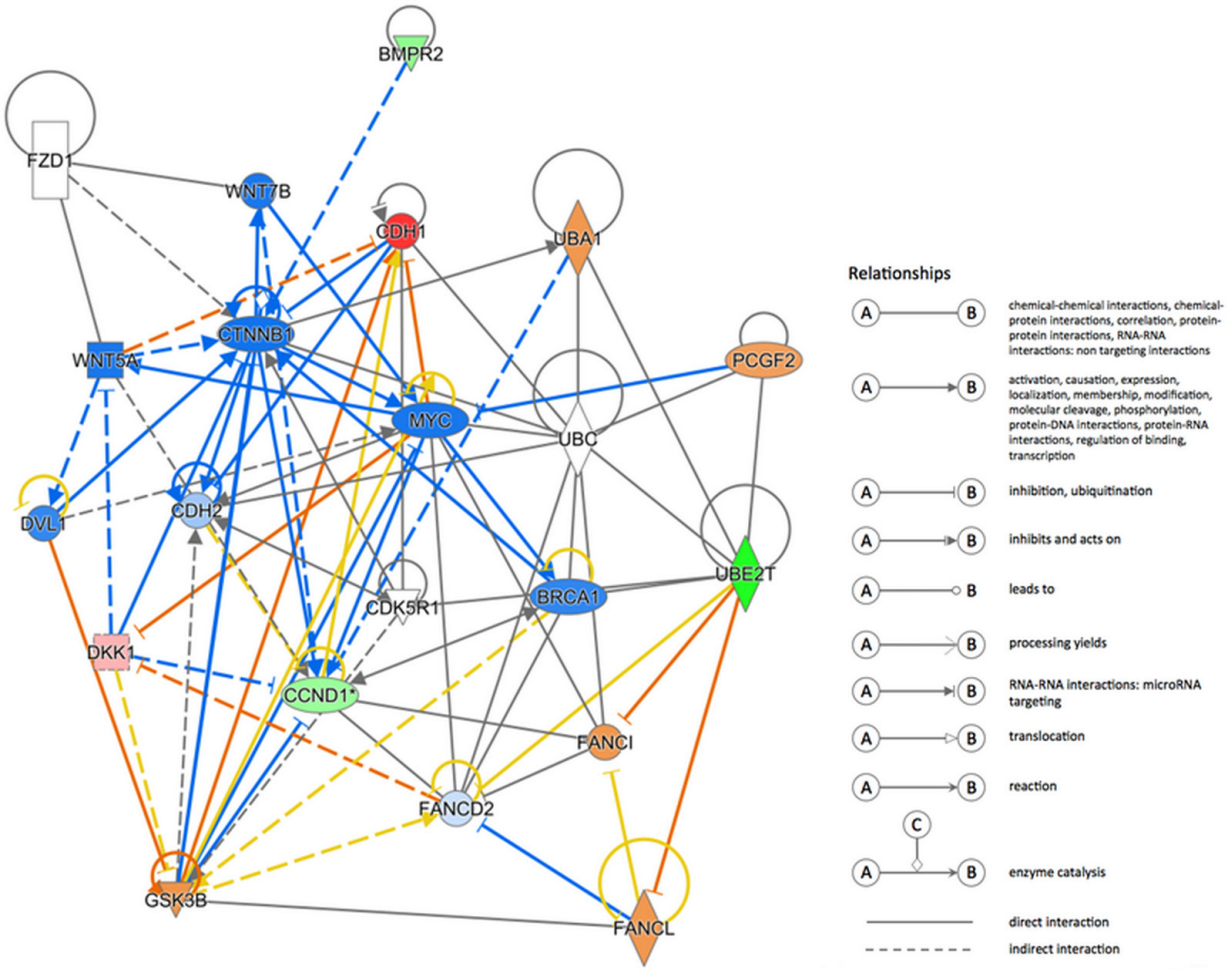
UBE2T regulation network A possible regulation network based on the results of this study and previous research is shown.

To explore the regulation of UBE2T in gastric cancer, path-array analysis was performed on 3 samples each from the NC and KD groups using gene chips. Sample quality was confirmed prior to the analysis. Correlations between samples in each group were analyzed; the associated Pearson's Correlation Signal values are shown in Figure 8A. A clustering dendrogram was used to identify differences in gene expression between the two groups. Compared to the control group, a total of 226 genes (shown in red) were up-regulated, while a total of 282 genes (shown in green) were down-regulated, in the KD group. Genes for which no obvious changes were observed are shown in black, and undetected genes are shown in gray. The results are shown in Figure 8B, shorter distances indicate a more similar relationship. Meta-analysis was performed to detect pathways that might be regulated by or related to UBE2T using IPA online software. The results suggested that many cellular events, such as growth and proliferation, movement, and cell death and survival, are involved in the regulation of UBE2T (Figure 8C). A possible regulation network for UBE2T based on our results and the results of previous studies is shown in Figure 9.

### Analysis of UBE2T-related signaling factors and networks

**Figure 10:**
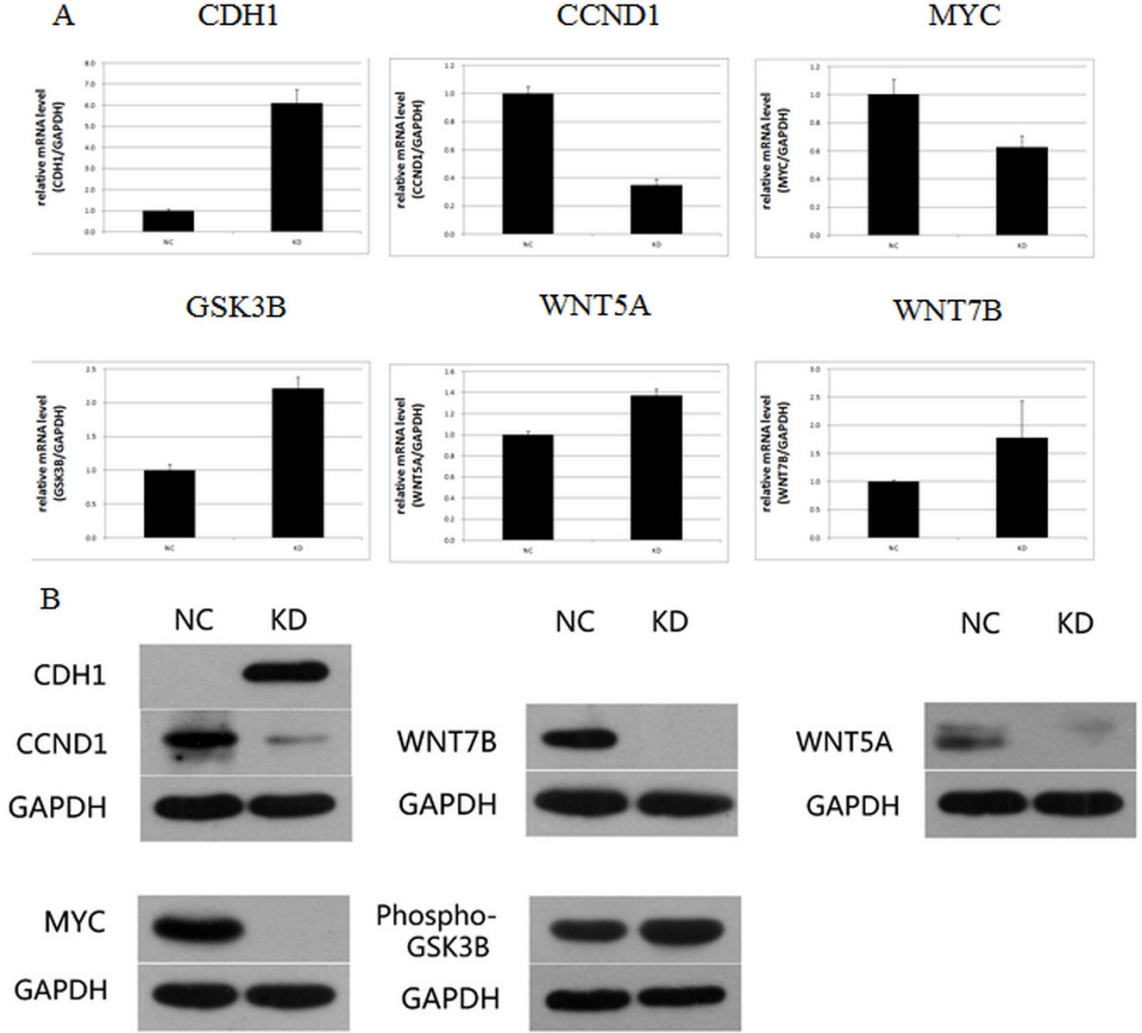
Signaling factors and networks related to the regulation of UBE2T (**A**) Expression of CDH1, CCND1, MYC, GSK3β, WNT5A, and WNT7B mRNA after UBE2T knockdown was analyzed using real-time PCR. Data are presented as means ± SD. (**B**) Expression of CDH1, CCND1, MYC, WNT7B, Phospho-GSK3B, and WNT5A proteins after UBE2T knockdown was analyzed using Western blots.

The expression of UBE2T-related factors identified in the path-array analysis was then analyzed at the gene level using real-time PCR and at the protein level using Western blots in tumor tissues from xenografted mice. As shown in Figure 10A, CDH1, Phospho-GSK3B, WNT7B, and WNT5A mRNA expression were increased in the KD group compared to the NC group. In contrast, CCND1 and MYC mRNA expression were decreased in the KD group. The Western blot results were similar to those obtained using RT-PCR. As shown in Figure 10B, WNT7B, MYC, and WNT5A protein expressions were decreased and almost undetectable in the NC group. Phospho-GSK3B protein expression also increased by 42.1%, in the KD group. In contrast, CDH1 protein expression, which was very weakly expressed in the NC group, increased in KD group. CCND1 protein expression also decreased by 85.2% in the KD group. Except for WNT7B and WNT5A, for which protein expression decreased although mRNA expression was unchanged, similar changes in expression were observed at both the mRNA and protein levels for all factors examined. Taken together, these results and previous research indicates that UBE2T might play a critical role in gastric cancer and might serve as useful potential biomarker and therapeutic target in gastric cancer patients.

## DISCUSSION

The eukaryotic ubiquitin-proteasome system (UPS) is responsible for the degradation of most intracellular proteins. Both monoubiquitination and polyubiquitination influence proteasomal protein degradation and are involved in many diseases [11–13], including gastrointestinal cancer [14]. Most studies have focused on mutations in or overexpression of numerous E3 ubiquitin ligases that result in tumor suppression [15–17], while few have examined the effects of ubiquitin-conjugating enzymes (E2 enzymes) such as UBE2T. In addition to its role in mitomycin-C (MMC)-induced DNA repair, UBE2T mediates monoubiquitination of FANCL and FANCI [3, 4]. UBE2T expression is elevated in human prostate cancer, breast cancer, and lung cancer [6–8]. However, few studies have examined UBE2T expression in gastric cancer, which is the number one cause of cancer-related death in China and a top cause of cancer-related death globally [9, 18].

In this study, we first examined UBE2T expression in gastric tumor and para-carcinoma tissues samples collected during surgical operations. UBE2T expression was increased in gastric tumors compared with para-carcinoma tissues. Similar results were obtained using samples from TCGA database. However, we did not find a clear link between UBE2T expression and tumor differentiation. UBE2T mRNA expression, which was measured in SGC-7901, MKN-45, MGC80-3, and BGC823 gastric cancer cells using real-time PCR, was high in all of the gastric cancer cells and positively correlated with measures of malignancy. However, other factors that contribute to malignant characteristics *in vivo* may complicate this relationship between UBE2T expression and malignancy in clinical samples.

Next, we examined whether siRNA-mediated suppression of UBE2T expression in SGC-7901 and BGC-823 cells inhibited gastric cancer progression. After confirming that siRNA successfully suppressed UBE2T expression, we measured cellular growth using MTT and colony formation assays and Cellomics detection. These three measurements, together with previous findings, indicate that suppression of UBE2T inhibits proliferation in cancer cells. The cell cycle, which is crucial for cell survival, proliferation, and growth, is a target of anti-cancer treatment [19]. FCM results revealed that suppression of UBE2T increased G2/M cell cycle arrest and promoted apoptosis in gastric cancer cells. Cell cycle arrest results in abnormal cell growth, which then triggers apoptosis; targeted gastric cancer therapies currently take advantage of this process [20].

The metastatic cascade includes many processes, the first of which is invasion. Many migration and invasion mechanisms, including both single and multi-cellular processes, have been identified and targeted by various cellular interventions [21]. Because AGS and SGC-7901 cells have shown elevated invasive and metastatic activity in previous studies, we examined these cells to determine the effects of UBE2T on invasive and metastatic ability in gastric cancer cells [22, 23]. Our results indicate that suppression of UBE2T attenuated cell migration and invasion, but not cell adhesion. Cancer cell adhesion is a complex process that involves many cellular interactions, modulation of cytoskeletal assembly, receptor-ligand binding, and intracellular signaling cascades [24]. Our findings suggest that UBE2T may have complex effects on cellular interactions; these effects should be examined in future studies. Epithelial–mesenchymal transition (EMT) is the process in which epithelial cells lose cell polarity and cellular adhesion, differentiate into other cell types, and migrate and invade distant tissues. In addition to its roles in mesoderm formation, neural tube formation, and wound healing, EMT is also crucial in the initiation of cancer metastasis and invasion. Factors that regulate cellular adhesion, including E-Cadherin, Fibronectin, Vimentin, and β-Catenin, play important roles in EMT. UBE2T knock-down increased E-Cadherin and β-Catenin expression and decreased Fibronectin and Vimentin expression, and these changes in expression likely inhibited metastasis and invasion in gastric cancer cells.

Together, these *in vitro* experiments suggest that suppression of UBE2T inhibits growth and colony formation and promotes apoptosis in gastric cancer cells. To confirm that these results were applicable *in vivo*, we used cancer cell xenotransplantation in nude female mice to examine the effects of UBE2T knockdown on gastric cancer progression. We found that gross tumor volumes and weights were lower in BGC-823 KD mice than in the NC group, suggesting that UBE2T knockdown also inhibited tumor formation and progression *in vivo*. Our results also indicated that invasive and metastatic abilities of the BGC-823 KD cells were clearly reduced in KD group mice. Together, these data indicated that UBE2T knockdown also attenuated gastric tumor formation and growth *in vivo*.

Next, we identified other factors and pathways that regulate or are regulated by UBE2T in gastric cancer using Affymetrix gene chip assays. In accordance with the *in vivo* and *in vitro* results, this analysis indicated that UBE2T was involved in cellular growth and proliferation, cellular movement, and cell death and survival. The predicted gene interactions shown were then confirmed using both real-time PCR and Western blots. Suppression of UBE2T increased the expression of CDH1 and Phospho-GSK3B. The classical cadherin CDH1 (also known as E-Cadherin) is processed to generate a mature calcium-dependent cell-cell adhesion protein comprised of five extracellular cadherin repeats, a transmembrane region, and a highly conserved cytoplasmic tail. Decreased CDH1 expression is positively correlated with gastric cancer progression [25–27]; the decreased CDH1 (E-Cadherin) expression observed here might partially explain the ability of UBE2T knockdown to attenuate gastric tumor formation and growth. GSK3B (glycogen synthase kinase 3 beta) is a serine-threonine kinase involved in energy metabolism, neuronal cell development [28], inflammation, and osteoporosis [29] that also plays an important role in transcriptional regulation and oncogenic signaling. Deregulated GSK-3β expression promotes gastrointestinal, pancreatic, and liver cancers and glioblastomas [30]. Inhibition of GSK-3β attenuates cell survival and proliferation, induces cell senescence and apoptosis, and sensitizes tumor cells to chemotherapeutic agents and ionizing radiation.

Suppression of UBE2T also decreased CCND1, Wnt5A, Wnt7B, and c-Myc expression. CCND1, which is required for progression through the G1 phase of the cell cycle, is rapidly synthesized during that phase and then degraded during S phase. CCND1 also dimerizes with cyclin-dependent kinases (CDK) 4/6 to regulate the G1-S phase transition and inhibits retinoblastoma protein (pRb) activity, and overexpression of CCND1 is associated with tumor progression in gastric and other cancers [31, 32]. Surprisingly, we found that UBE2T knockdown resulted in cell cycle arrest at the G2/M phase rather than the G1/S phase. In addition to CDK, CCND1 binds to nuclear receptors such as estrogen receptor α, thyroid hormone receptor, PPARγ, and AR, as well as to histone acetylases and histone deacetylases, to regulate cell proliferation, growth, and differentiation [33, 34]. It is possible that that UBE2T knockdown-induced inhibition of CCND1 preferentially affects these activities, which may be independent of CDK-dependent cell cycle arrest at the G1-S phase. As members of Wnt family, both Wnt5A and WNT7B play essential roles in regulating developmental pathways during embryogenesis and oncogenesis [35].

Wnt proteins increase nuclear and cytoplasmic β-catenin expression, in turn activating the transcription of CCND1 and c-myc [36]. In cancer, Wnt is also involved in EMT [37, 38]. Suppression of UBE2T inhibited Wnt5A and WNT7B, thus inhibiting tumor growth. c-Myc, a well-known oncogene, is overexpressed in many types of human cancer, including gastric cancer, breast cancer, and lung cancer, and its activity is regulated by phosphorylation, acetylation, and ubiquitylation [39]. Recently, it was discovered that c-Myc is a target of SUMOylation-dependent proteasomal degradation, in which UBE2T plays an important role [40]. Here, suppression of UBE2T inhibited the ubiquitin-proteasome pathway and c-Myc expression, thereby inhibiting tumor growth. Taken together, these data suggest that suppression of UBE2T first inhibits cell cycle progression by inhibiting CCND1, which greatly reduces cellular proliferation. Suppression of UBE2T also inhibits gastric tumor invasion and metastasis via the WNT signal pathway by inhibiting WNT5A and WNT7B, which in turn promotes GSK3B expression, activates β-Catenin, and inhibits the key oncogene c-Myc. In addition, the UBE2T knockdown-induced increase in CDH1 (E-Cadherin) also inhibits gastric tumor formation and growth. Together, all of these factors contribute to the UBE2T knockdown-induced inhibition of progression in gastric cancer both *in vitro* and *in vivo*. These results suggest that UBE2T may serve as a useful prognostic biomarker and therapeutic target in gastric cancer patients.

## MATERIALS AND METHODS

### Immunohistochemical detection of UBE2T in gastric tumors and para-carcinoma tissues

Informed consent was obtained from all patients involved in this study, which was conducted according to the guidelines and with the approval of the Medical Ethics Committee of Lanzhou University Second Hospital. Sections of gastric tumors and para-carcinoma tissues (4 μm thick) were washed in xylene for 15 min two times, followed by sequential 10 min washes as follows: 50% xylene/50% ethanol mixture, 100%, 95%, 85%, and 75% ethanol, ddH_2_O, and 3% H_2_O_2_. After antigen retrieval, sections were blocked in 10% serum for 30 min, incubated in a humidified chamber overnight with Anti-HSPC150 /UBE2T Antibody (Abcam, 1:100) at 4°C, and then incubated with an UltraSensitiveTM SP kit (Fuzhou Maixin Biotech. Co., Ltd.) in a humidified chamber for 1h at room temperature (RT). After DAB and hematoxylin staining, photos were taken under an inverted microscope (XDS-100, Shanghai Cai Kang Optical Instrument Co., Ltd.).

### TCGA data analysis of UBE2T expression in different gastric cancer cells

UBE2T RNA-seq data for a total of 29 samples in TCGA database were analyzed using edgeR software. *P* values were calculated according to the generalized linear models. SGC-7901, MKN-45, MGC80-3, BGC-823, and AGS gastric cancer cells were cultured at the Key Laboratory of Digestive System Tumors of Gansu Province. The following UBE2T and GAPDH primers were designed using Beacon Designer 2 software and synthetized by Shanghai GeneChem Co., Ltd: UBE2T: F: TTGATTCTGC TGGAAGGATTTG, R: CAGTTGCGATGTTGAGGGAT, product length: 84 bp; GAPDH: F: TGACTTCAACAGCGA CACCCA; R: CACCCTGTTGCTGTAGCCAAA, product length: 121 bp. Real-time qPCR consisted of an initial 15 sec hold at 95.0°C followed by 45 cycles of 95.0°C for 5 sec and 60.0°C for 30 sec. Data collection and real-time analysis were enabled. Melt curve detection parameters were as follows: initial 1 min hold at 95.0°C, second 1 min hold at 55.0°C, and 4 sec holds at 55.0°C–95.0°C for 81 cycles with the set temperature increasing by 0.5°C per cycle after cycle 2. Data were analyzed using the 2^−ΔΔCt^ method.

### siRNA transfection

The target sequence for the UBE2T siRNA was GTACACAACTCAACACAGAAA. pGCSIL-GFP was used as vector and was double digested using Age I and EcoR I. After PCR identification, SGC-7901 and BGC-823 cells were infected with positive lentiviral vectors in DMEM medium plus 10% FBS with Eni.S + polybrene at a titer of 3 × 10^8^ TU/mL. Human gastric carcinoma cells were cultured in 1640 medium plus 10% FBS. 13.3 μl LV-UBE2T-RNAi (41421-1) viruses with title at 3 ×10^8^TU/ml were used for KD cells, while 8 μl psc3741 virus with title at 5 × 10^8^ TU/ml was used for the negative controls. Puromycin at 4 μg/mL was added to the medium 96 h after infection, followed by 48 h of incubation. Fluorescent pictures were taken 96 h after infection.

### Cellular proliferation assay

SGC-7901 and BGC-823 cells infected with shUBE2T or shCtrl were plated in a 96-well plate at a density of 2000 cells in 100 μL per well and placed in a culture incubator at 37°C and 5% CO_2_. Cellomics detections were performed every day for a total of 5 days and were used to draw cellular proliferation curves. Cell proliferation rates were determined using MTT. At the indicated time points, MTT solution was added to each well followed by incubation for 4 hours. Absorbance values were obtained at 490/570 nm. For the colony formation assay, 800 shUBE2T or shCtrl cells per well were seeded in 6-well tissue culture plates. Fourteen days after incubation, cell colonies were fixed with 4% PFA and stained with Giemsa staining. Colonies with more than 50 cells were counted using a light microscope.

### Cell cycle and apoptosis detection

Cell cycle and apoptosis detection were performed using flow cytometry (FCM). Cells were harvested by centrifuging at 1500 rpm for 5 min, fixed with ethanol for 1 h at 4°C, and stained with PI. Cells were analyzed using FCM at a speed of 200-350 cells/s. The percentage of cells in each cell cycle phase was analyzed. To measure apoptosis, cells were harvested by centrifuging at 1500 rpm for 5 min and washed with 1 × binding buffer and 1× staining buffer. 5 μL of annexin V-APC staining solution was added to 100 μL of cell suspension in the dark and then detected via FCM.

### Cell scratch assay

Migration ability was assessed using a cell scratch assay. A total of 3 × 10^4^ cells in the logarithmic phase were seeded into 96-well cell culture plates and cultured to 90% confluence. Three parallel, linear wounds were then generated in each dish using a Micro Scratch Tester (MST). After an additional 8 and 24 hours of culture, migration was assessed. Three representative photos of the scratched areas from each dish were taken using a microscope and analyzed.

### Trans-well invasion assay

A matrix-coated trans-well invasion assay was performed to measure invasive ability. Briefly, 1 × 10^5^ cells in the logarithmic phase were suspended in 500 μL of cell culture medium and added to the top chamber of a matrix-coated 24-well plate (Corning, Cambridge, MA, USA). The cells were then incubated for 24 h in a 37°C cell incubator. 5 × 10^3^ cells were cultured in another 96-well plate as control. The membranes were then fixed and stained using Giemsa solution for 3 min. After dislodging cells remaining on the membrane, the numbers of cells below the membrane were counted.

### Cell adhesion test

2 μg/50 μL Matrigel was added to a 96-well plate and dried. 1× PBS was used to wash excess gel from the plate. Four thousand cells in the logarithmic phase were seeded on a Matrigel plate for 40 min in a 37°C cell incubator. The culture solution and unadhered cells were then removed. Cell numbers were counted using Celigo scanning.

### Generation of xenotransplantation gastric tumor model

This experiment was conducted in accordance with Animal Welfare and 3Rs (Replacement, Reduction, and Refinement) guidelines and was approved by the Laboratory Animal Ethics Committee and Medical Ethics Committee of Lanzhou University Second Hospital. Female nude mice (4 weeks old, BALB/c background, average weight: 18 g) were purchased from Shanghai Linchang Biotech Co., Ltd., and used to generate the xenotransplantation tumor model. Human gastric carcinoma cells BGC-823 cells were micro-injected at a density of 4 × 10^6^ into the right upper limbs of these mice to generate NC and KD groups with 10 mice each. Tumor sizes were measured on the 7th, 11th, 13th, 15th, and 18th days after transplantation. Tumor size was calculated as V = π/6 × L × W^2^ (L: major axis; W: minor axis). No tumor was larger 2000 mm^3^. All mice were sacrificed on the 18th day and tumors were collected and weighted.

### Path-array analysis

Gene chips were used for path array analysis and meta-analysis with IPA online software. Signal Histogram, Relative Signal Box Plot (data not shown), and Pearson's correction signal confirmed the quality of these analyses. Classical pathways and related diseases were analyzed using IPA (www.ingenuity.com). More detailed information and instructions are provided at www.ingenuity.com.

### Real-time PCR and western blot detection

Real-time PCR was performed as previously described. The primers used and associated product lengths are shown in Table 3. Total proteins extracted from samples were quantified using the BCA kit. Twenty μg of protein were loaded in SDS-PAGE gels. After electrophoresis, proteins were transferred to PVDF membranes, blocked, incubated with primary antibodies at 4°C overnight, and then incubated with secondary antibodies for 1 h at room temperature. Bands were visualized using enhanced chemiluminescence (ECL) reagents. All data were analyzed using gray density scanning and comparison methods. GAPDH was used as a control for both real-time PCR and Western blots. The primary antibodies used are listed in Table 4.

**Table 3:** The primers and length of products of relative genes

Target gene	F primer 5′-3′	R primer 5′-3′	length of product (bp)
GAPDH	TGACTTCAACAGCGACACCCA	CACCCTGTTGCTGTAGCCAAA	121
WNT7B	TCCACTGGTGCTGCTTCG	GTCACGGGTGCTGTTCTGC	300
GSK3B	GCACTGTGTAGCCGTCTG	GAGGAGGAATAAGGATGGTAGC	199
CDH1	AACGCATTGCCACATACA	CGGGCTTGTTGTCATTC	114
WNT5A	TCGACTATGGCTACCGCTTTG	CACTCTCGTAGGAGCCCTTG	84
CCND1	GGTGGCAAGAGTGTGGAG	CCTGGAAGTCAACGGTAGC	148
MYC	TGTCCGTCCAAGCAGAGG	CGCACAAGAGTTCCGTAGC	107

**Table 4:** Primary antibodies used for the protein expression detection

Protein Detection	Sources of Host	Company Name	Dilution as primary Ab
E-Cadherin	Mouse	abcam	1/500
Fibronectin	Mouse	abcam	1/500
Vimentin	Rabbit	CST	1/500
β-Catenin	Rabbit	CST	1/500
Wnt7b	Rabbit	abcam	1 μg/ml
CDH1	Mouse	abcam	appropriate
CCND1	Rabbit	CST	1/1000
c-Myc(MYC)	Rabbit	abcam	1/10000
Phospho-GSK3B	Rabbit	CST	1/1000
Wnt5a	Rabbit	CST	1/1000
GAPDH	Mouse	Santa-Cruz	1/5000

### Statistical analysis

All data are expressed as means ± SD and were analyzed using SPSS 16.0 (Chicago, IL, USA). One-way ANOVAs were used for multivariate analyses and student's *t*-tests were used for univariate analyses. Statistical significance was defined as *P* < 0.05(*), and differences with *P* < 0.01(**) were considered highly significant. Data analysis for IHC is listed in Tables 1 and 2.
