# Correction: UBE2T knockdown inhibits gastric cancer progression

**DOI:** 10.18632/oncotarget.28356

**Published:** 2023-03-11

**Authors:** Changjiang Luo, Yunyi Yao, Zeyuan Yu, Huinian Zhou, Lingyun Guo, Junqiang Zhang, Hongtai Cao, Genyuan Zhang, Yumin Li, Zuoyi Jiao

**Affiliations:** ^1^Department of General Surgery, Lanzhou University Second Hospital and Key Laboratory of Digestive System Tumors of Gansu Province, Lanzhou, Gansu, 730030, China; ^2^Department of Medical Technology and Key Laboratory of Biotechnology for Laboratory Medicine of Suzhou, Suzhou Vocational Health College, Suzhou, Jiangsu, 215009, China; ^*^These authors contributed equally to this work

**This article has been corrected:** In Figure 4C, in the SGC-7901 panel, the first image in the first row is an accidental duplicate of the third image in the second row. The corrected Figure 4, obtained using the original data, is shown below. The authors declare that these corrections do not change the results or conclusions of this paper.

Original article: Oncotarget. 2017; 8:32639–32654. 32639-32654. https://doi.org/10.18632/oncotarget.15947

**Figure 4:**
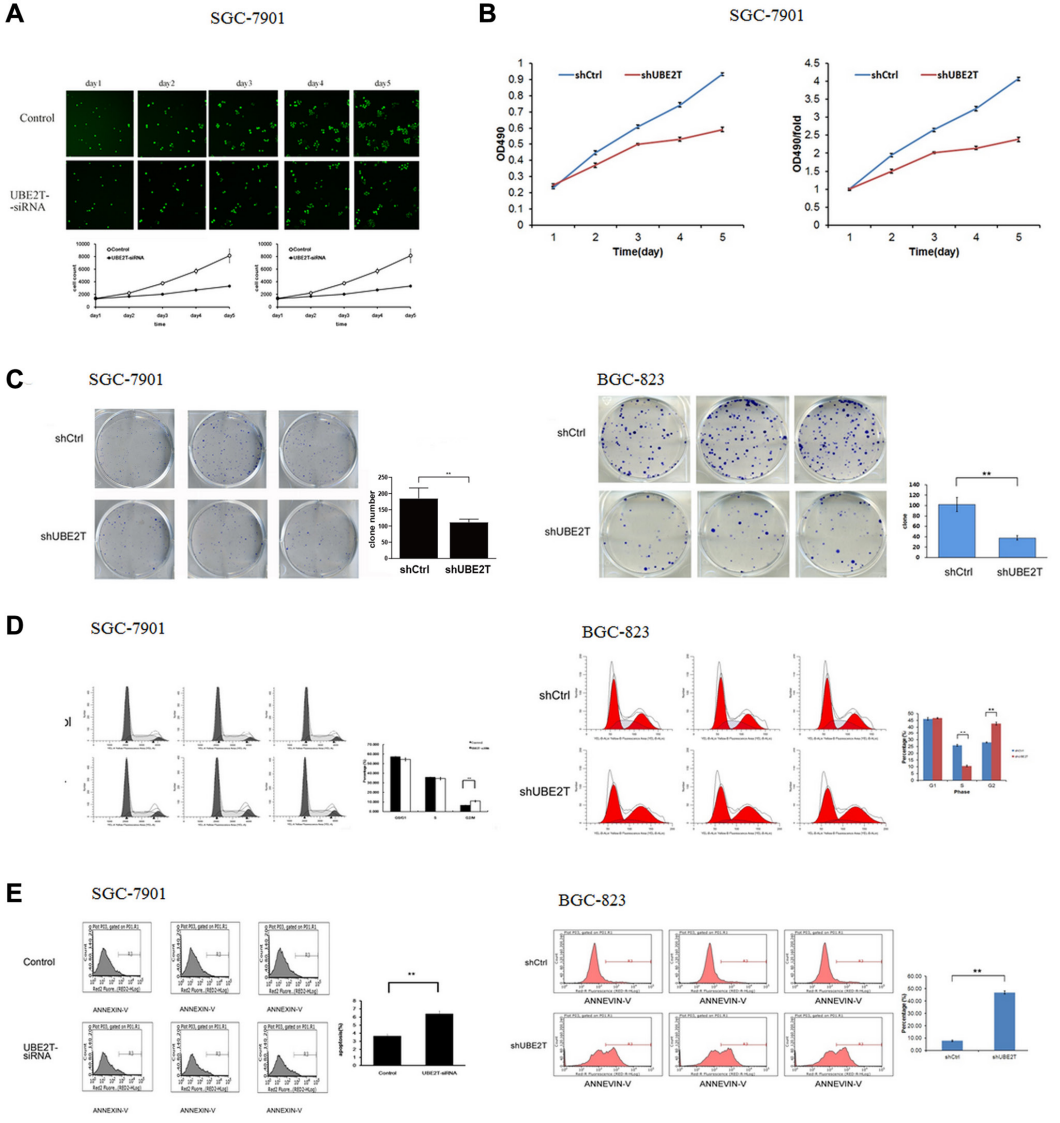
Suppression of UBE2T inhibits growth and colony formation in gastric cancer cells. (**A**) Cellomics detection indicated that SGC-7901 cell proliferation decreased after suppression of UBE2T. Data are presented as means ± SD. (**B**) MTT assays in SGC-7901 cells indicated that suppression of UBE2T inhibited SGC-7901 cell proliferation. Data are presented as means ± SD. (**C**) The tumor colony formation assay indicated that suppression of UBE2T inhibited tumor colony formation in SGC-7901 and BGC-823 cells. Data are presented as the means ± SD. ^**^ *P* < 0.01 versus the control group. (**D**) The percentages of cells in the G1, S, and G2/M phases were determined using FCM. G1 and S phase populations decreased while the G2/M phase population increased in SGC-7901 and BGC-823 cells. Data are presented as means ± SD. ^**^ *P* < 0.01 versus the control group. (**E**) Annexin V staining was measured using FCM to evaluate apoptosis. The percentage of apoptotic SGC-7901 and BGC-823 cells increased dramatically after siRNA lentivirus infection. Data are presented as means ± SD. ^**^ *P* < 0.01 versus the control group.

